# Plasma Proteomics Enable Differentiation of Lung Adenocarcinoma from Chronic Obstructive Pulmonary Disease (COPD)

**DOI:** 10.3390/ijms231911242

**Published:** 2022-09-24

**Authors:** Thilo Bracht, Daniel Kleefisch, Karin Schork, Kathrin E. Witzke, Weiqiang Chen, Malte Bayer, Jan Hovanec, Georg Johnen, Swetlana Meier, Yon-Dschun Ko, Thomas Behrens, Thomas Brüning, Jana Fassunke, Reinhard Buettner, Julian Uszkoreit, Michael Adamzik, Martin Eisenacher, Barbara Sitek

**Affiliations:** 1 Clinic for Anesthesiology, Intensive Care and Pain Therapy, University Medical Center Knappschaftskrankenhaus Bochum, 44892 Bochum, Germany; 2 Medizinisches Proteom-Center, Ruhr-University Bochum, 44801 Bochum, Germany; 3 Center for Protein Diagnostics (PRODI), Medical Proteome Analysis, Ruhr-University Bochum, 44801 Bochum, Germany; 4 Institute for Prevention and Occupational Medicine of the German Social Accident Insurance, Institute of the Ruhr University Bochum (IPA), 44789 Bochum, Germany; 5 Department of Internal Medicine, Johanniter-Kliniken Bonn GmbH, Johanniter Krankenhaus, 53113 Bonn, Germany; 6 Institute of Pathology, Medical Faculty and Center for Molecular Medicine (CMMC), University of Cologne, 50924 Cologne, Germany

**Keywords:** lung cancer, plasma proteomics, machine learning, artificial intelligence, random forest, Ig kappa light chain, SERPINA3, SAA1

## Abstract

Chronic obstructive pulmonary disease (COPD) is a major risk factor for the development of lung adenocarcinoma (AC). AC often develops on underlying COPD; thus, the differentiation of both entities by biomarker is challenging. Although survival of AC patients strongly depends on early diagnosis, a biomarker panel for AC detection and differentiation from COPD is still missing. Plasma samples from 176 patients with AC with or without underlying COPD, COPD patients, and hospital controls were analyzed using mass-spectrometry-based proteomics. We performed univariate statistics and additionally evaluated machine learning algorithms regarding the differentiation of AC vs. COPD and AC with COPD vs. COPD. Univariate statistics revealed significantly regulated proteins that were significantly regulated between the patient groups. Furthermore, random forest classification yielded the best performance for differentiation of AC vs. COPD (area under the curve (AUC) 0.935) and AC with COPD vs. COPD (AUC 0.916). The most influential proteins were identified by permutation feature importance and compared to those identified by univariate testing. We demonstrate the great potential of machine learning for differentiation of highly similar disease entities and present a panel of biomarker candidates that should be considered for the development of a future biomarker panel.

## 1. Introduction

Lung cancer is the leading cause of death in all cancer types and makes up about 18% of cancer-related deaths worldwide while contributing to about 11% of all diagnosed cancer cases [1,2]. The main cause for lung cancer is smoking [3], which is associated with more than 90% of all lung cancer cases [2]. Smoking is also a major risk factor in the development of a plethora of other diseases related to the respiratory tract, especially lung cancer and COPD. Chronic obstructive pulmonary disease (COPD) develops in about 50% of smokers over time [4]. Smokers with COPD have a two- to five-times higher risk of developing lung cancer [5,6] and COPD is found in about 50–90% of lung-cancer patients [7]. Five-year survival rates for lung cancer are low with about 20%, reflecting the large number of diagnoses at late stages, when 57% of the patients already show metastatic progress. For patients presenting with metastatic disease, the 5-year survival rate is only 5% in comparison to 57% for localized stages [8]. Due to the fatality of lung cancer and the high risk of COPD patients for developing AC, biomarkers for detection of AC and differentiation from COPD are urgently needed, preferably in body fluids that can be obtained by minimal-invasive methods. Currently, diagnosis is commonly performed via invasive biopsy, mostly after observation of visible changes in CT scans or bronchoscopy.

Mass-spectrometry (MS)-based proteomics is a powerful tool for high-throughput discovery approaches in the area of biomarker research. A wide range of materials can be used for analysis including serum or plasma, which, in addition to tissue, are major sources for biomarker discovery. Minimally- or noninvasive biomarkers would improve cancer assessment as diagnostic or prognostic markers, as well as a tool for monitoring [9]. Numerous proteomics studies analyzing tissue, bronchoalveolar lavage fluid, and blood have been performed for identifying biomarkers for AC, COPD, and the differentiation of both [10]. Several candidate biomarkers have been proposed, including AGR2 [11,12], SAA [11], HER2 [11,13,14], APOE [15], or SCGB3A2 [16] for AC; YKL-40 [17], MFAP4 [18], GRP78, soluble CD163, IL1AP, and MSPT9 for COPD [11]; CRP, VEGF [13,14,19], IL-8, and MMP9 [14] for the differentiation of AC and COPD. However, none of those proposed biomarker candidates have yet taken the step into clinical practice; therefore, reliable biomarkers for these applications are still highly demanded.

With the rise of a multitude of bioinformatics methods, machine learning approaches for the separation of disease groups became applicable to a larger life science community to aid in the identification of protein candidates [20]. Logistic regression and linear discriminant analysis (LDA) are classical modeling statistics. Logistic regression and LDA are easy to interpret with regard to the influence of the predictor variables, but can only perform a linear separation of the data. The support vector machines (SVMs) [21] can use transformation in a higher-dimensional space by the use of a polynomial kernel function to learn more complex (separation) patterns than logistic regression or LDA. Random forest is based on tree-building algorithms [22] and is robust against overfitting. All the stated approaches can be used for large numbers of predictor variables in limited sample sizes and with heterogeneous data, making them ideal tools for clinical proteomics data analysis.

**Figure 1:**
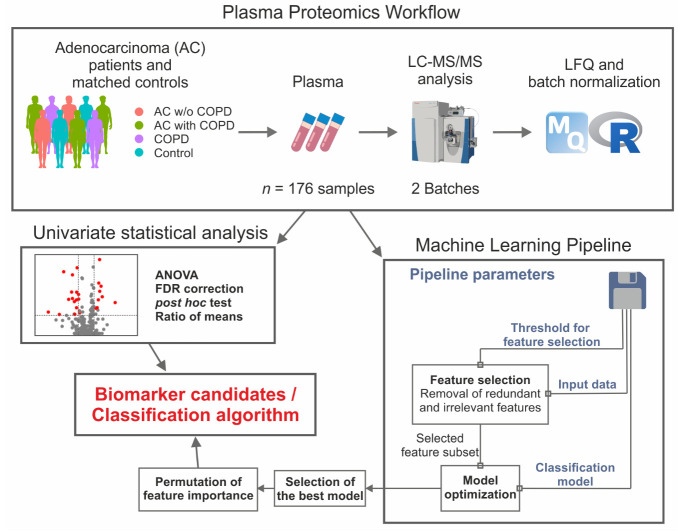
Schematic representation of the project workflow. Plasma samples from n = 176 patients were analyzed in two batches using LC–MS/MS. Proteins were quantified label-free and the resulting intensities were normalized to account for batch effects. Normalized intensities were analyzed by univariate statistics and machine learning approaches. Five different machine learning algorithms were compared using the same modeling pipeline.

In this study, we used mass spectrometry analysis of plasma samples for the identification of biomarker candidates using univariate statistics and machine-learning-based classification algorithms. We analyzed samples of AC patients with or without COPD, COPD patients, and hospital controls (HCs) with the aim of identifying proteins differentiating AC from COPD (Figure 1). We developed classification models to *(A)* separate all AC patients from COPD patients (AC vs. COPD), and *(B)* specifically differentiate AC patients with a COPD background from COPD patients (AC with COPD vs. COPD). We optimized the feature selection and tested several machine learning algorithms for classification. After choosing the best model, we calculated the permutation feature importance to determine the most influential proteins. To further validate the results of the model, we repeated the analysis in 50 random train-test-splits of the dataset for model *(A)* and compared the precision on the test sets as well as the most often used features to the model with the full dataset. This allowed us to compare the results of univariate tests and machine learning. We demonstrated that plasma proteomics is a powerful technique to distinguish patient groups that are challenging to discriminate in clinical routine. We identified a set of proteins using complementary approaches, which might serve as a starting point for the development of a clinical biomarker panel for diagnosis and differentiation of AC and COPD.

## 2. Results

### 2.1. Successful Normalization of Label-Free Proteomics Data

**Figure 2:**
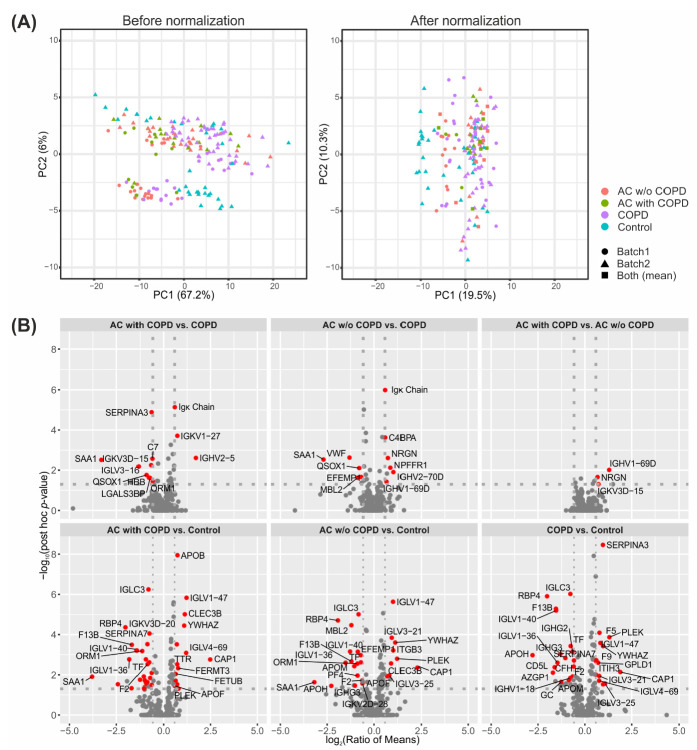
Normalization and statistical analysis of proteomics data. (**A**) Principal component analysis (PCA) plots of label-free LC–MS/MS data before and after batch normalization. Each data point corresponds to a sample measured in either batch 1, batch 2, or both batches (colors representing patient groups). (**B**) Volcano plots representing the results of statistical analysis using Welch-ANOVA. Significant proteins highlighted in red (ANOVA *p*_FDR_-value ≤ 0.05 (corrected according to Benjamini–Hochberg); post hoc *p*_FDR_-value ≤ 0.05 (corrected according to Bonferroni–Holm); absolute ratio of means ≥ 1.5) and labeled with gene names (except Igκ Chain).

Due to the reception of clinical samples at different time points, the 176 clinical plasma samples were analyzed in two separate batches, with two different instruments and different LC gradients. Principal component analysis (PCA) of nonnormalized intensities showed two major clusters, which, however, did not represent the two analyzed batches (Figure 2A). The exact technical parameters that were responsible for the clustering of the data were unfortunately not identified. The applied normalization method keeps connections to biological effects while reducing systematic and technical errors. After normalization, samples of the two batches were much more congruent, which was highlighted by the reduced influence of PC1 (67.2% before normalization, 19.5% after normalization). Normalization was further evaluated using boxplots (Supplementary Figure S1) and MA plots (Supplementary Figure S2). Boxplots showed a decrease in inter-sample variability and MA plots showed good agreement between the samples that were doubly measured in both batches. The normalized protein intensities can be found in Supplementary Table S2.

### 2.2. Univariate Statistics Reveal Proteins Discriminating AC and COPD

**Figure 3:**
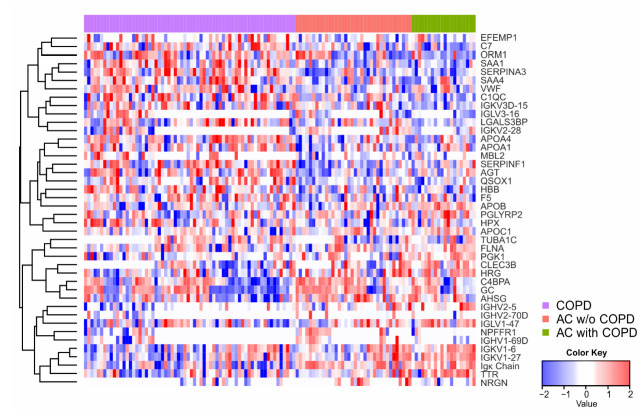
Hierarchical cluster analysis and two-group comparison of proteomics data. Heatmap illustrating hierarchical cluster analysis (distance based on Pearson’s correlation, complete linkage) considering 42 proteins, which passed a *p*_FDR_-value threshold ≤0.05 calculated using Welch-ANOVA for comparisons between either AC with or without COPD vs. COPD.

The combination of both analyzed batches led to the quantification of 397 protein groups in plasma. Of these, 83 protein groups were exclusively measured in batch 1, whereas 55 protein groups were exclusive for batch 2. At first, we analyzed the univariate protein differences between patient groups by means of ANOVA and the post hoc test. The statistical analysis showed the overall greatest differences for the comparisons between the diseased patient groups and hospital controls (Figure 2B, lower panel). This illustrates the need for appropriate control groups in clinical proteomics as the use of an unspecific control group—in this case, hospital controls—leads to many false findings. A maximum of 39 significantly differentially abundant proteins was observed for AC with COPD vs. Control (Table 1). In the following, the comparisons between the AC and COPD patient groups were emphasized, considering their clinical relevance. Here, both comparisons of AC with or w/o COPD vs. COPD resulted in 11 significantly differentially abundant proteins of which, however, only three proteins were regulated in both comparisons (Sulfhydryl oxidase 1 (QSOX1), Serum amyloid A-1 protein (SAA1), and Ig kappa light chain (no gene name; Supplementary Tables S3 and S4)). The comparison between AC with COPD and AC w/o COPD resulted in three significantly altered proteins. We performed hierarchical cluster analysis based on 42 proteins that were found with a significant *p*_FDR_ value between both AC groups and COPD by ANOVA. In addition to remarkable sample heterogeneity, the corresponding heatmap showed two major protein clusters separating COPD and AC (Figure 3).

### 2.3. Machine Learning Yields Highly Predictive Classification Models

**Figure 4:**
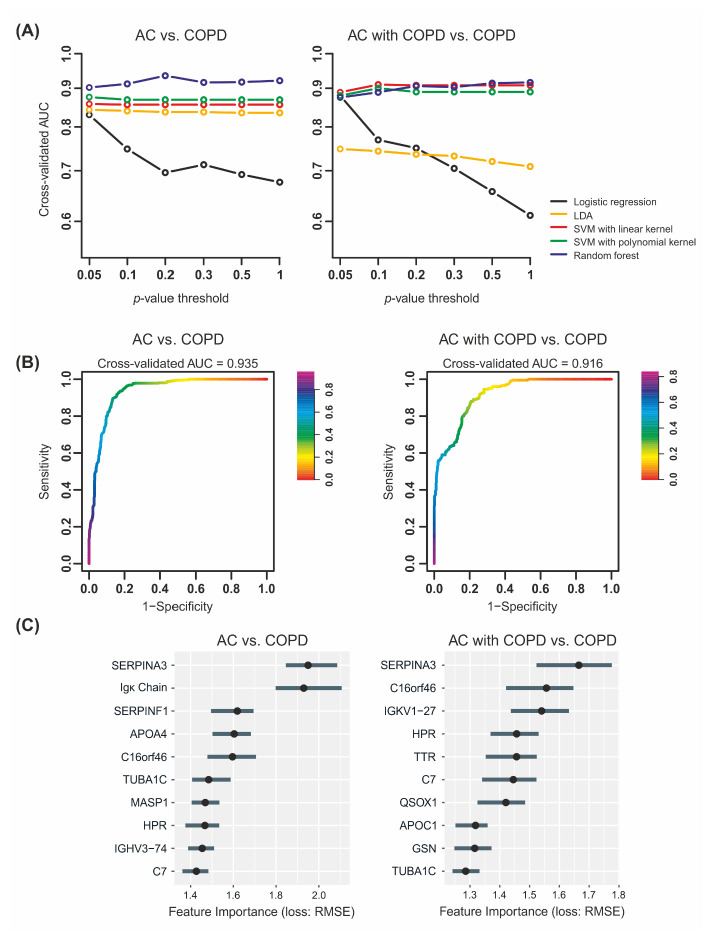
Results of machine learning approaches. (**A**) Five machine learning approaches were compared for classification of AC vs. COPD (left panel) and AC with COPD vs. COPD (right panel). Different *p*-value thresholds were assessed for feature selection and plotted against the respective ten-times-repeated 10-fold-cross-validated AUCs. (**B**) Receiver operating characteristic (ROC) curves for the best-performing random forest classifiers (AC vs. COPD: *p*-value threshold = 0.2; AC with COPD vs. COPD: no *p*-value threshold). (**C**) Feature importance plots illustrating the relative influence of individual proteins on multivariate classification models. Proteins represented by gene names (except Igκ Chain).

Five classification algorithms were assessed for the correct classification of *(A)* AC vs. COPD and *(B)* AC with COPD vs. COPD. For feature selection, increasing *p*-value thresholds were tested, resulting in increasing numbers of proteins considered for model optimization. Accordingly, the numbers of considered proteins were lowest for a *p*-value threshold of 0.05 with 24 proteins *(A)* and 12 proteins *(B)*. Without any cut-off, 194 proteins were considered for both *(A)* and *(B)*. For the differentiation of *(A)* AC vs. COPD, random forest classification outperformed the other models, achieving the maximum AUC when a *p*-value threshold of 0.02 was applied (Figure 3, Supplementary Table S5). The differences between individual *p*-value thresholds were small, however, and all models resulted in AUCs over 0.9. The highest AUC was 0.935 with a sensitivity of 0.848 and a specificity of 0.879 (Figure 4B, Table 2). The other classification models showed AUCs between 0.8 and 0.9 with SVM with the polynomial kernel, which is superior to SVM with the linear kernel and LDA. Logistic regression was outperformed by all models and showed a constant decrease in AUCs with increasing *p*-value threshold.

**Figure 5:**
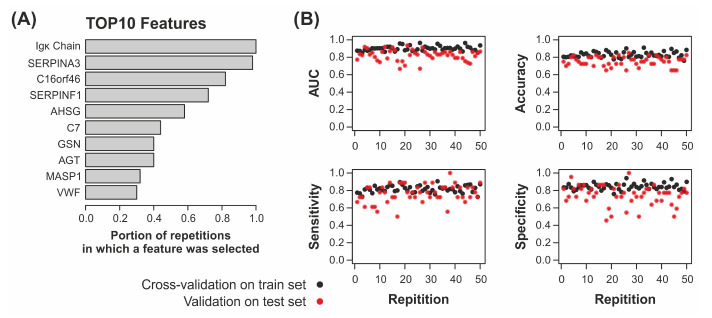
Results of intra-set validation for the comparison AC vs. COPD. The dataset was randomly split into train and test sets for 50 repetitions. The random forest model was developed with a ten-times-repeated 10-fold-cross-validation on the train set and validated on the test set. (**A**) Top 10 list of the most frequently selected features (i.e., proteins, represented by gene names (except Igκ Chain)). (**B**) Characteristics of the random forest classifier for cross-validation on the train sets (black) and validation on the test sets (red), respectively, were plotted against the number of repetitions.

For the differentiation of *(B)* AC with COPD vs. COPD, random forest and both SMV algorithms performed very similarly. Notably, SVM with the linear kernel performed better than random forest for some tested *p*-value thresholds (Figure 4A, Supplementary Table S5). The maximum AUC, however, resulted from random forest classification without feature selection by a statistical test (AUC = 0.916, sensitivity = 0.57, specificity = 0.965; Figure 4B, Table 3). Notably, LDA performed significantly poorer than the other models and logistic regression showed a striking performance decrease with increasing *p*-value thresholds. The subsequent feature importance analysis of the best-performing models revealed the greatest influence for Alpha-1-antichymotrypsin (SERPINA3) in both comparisons (Figure 4C). For *(A)*, Ig kappa light chain was the second-most informative feature followed by Pigment epithelium-derived factor (SERPINF1), Apolipoprotein A-IV (APOA4), and the uncharacterized protein C16orf46. For *(B)*, the feature importance was generally lower with SERPINA3 followed by C16orf46, Ig kappa variable 1–27 (IGKV1–27), Haptoglobin-related protein (HPR), and Transthyretin (TTR). Additional results from train-test-set validation for analysis *(A)* supported these results with mean AUC values of 0.823 over 50 repetitions on the test set and 0.901 in the cross-validation of the trainsets (Table 3, Figure 5). SERPINA3 and Igκ Chain were selected in 49 and 50 repetitions, respectively; SERPINF1 and C16orf46 were used in over 70% of the repetitions supporting the significance of these features. On the contrary, APOA4 was only used in three repetitions, but AHSG, which was not selected by the model for the full dataset, was selected in 29 (58%) repetitions.

### 2.4. Univariate Statistics and Machine Learning Reveal Candidates for a Biomarker Panel

The analysis of plasma proteomics data using univariate statistics revealed proteins that were significantly regulated between the AC subgroups with and without COPD, and COPD. The overlap between these proteins was small, illustrating the differences between the AC subgroups and the necessity of their individual consideration. Taken together, univariate analyses led to a comprehensive picture of protein regulations between the individual patient (sub-)groups and a list of robustly regulated proteins. The feature importance analysis of the best performing classification model resulted in complementary lists of candidates. Here, the most informative feature for both comparisons was Alpha-1-antichymotrypsin (SERPINA3), which was also significantly regulated in AC with COPD vs. COPD. Igκ Chain, which was regulated in both comparisons of AC subgroups vs. COPD, was also found to be highly informative using machine learning. These two features were also selected in 49 and 50 repetitions of the intra-set validation for AC vs. COPD. Further highly influential candidates were C7 and QSOX1, which were also identified by univariate testing. For univariate testing, a RoM threshold was applied to select candidates with distinct changes in intensities. For machine learning, no such filter was applied and, without considering the RoM threshold, additional proteins overlapped between both lists (i.e., SERPINF1, TTR, TUB1C, APOA4, and APOC1; Supplementary Table S3). Interestingly, C16orf46 and HPR, which were included in both random forest models, were not found to be significantly regulated in any univariate comparison. C16orf46 was selected in 41 of 50 repetitions during intra-set validation for AC vs. COPD.

## 3. Discussion

Identification of diagnostic biomarkers, especially in easily accessible body fluids such as blood, is the ultimate aim of clinical biomarker research. While the idealized concept of a single biomarker promises to be easily applicable, biomarker panels might realistically achieve higher precision, also taking into account the heterogeneity of the disease. On the pre-clinical level, mass spectrometry offers multiplex analysis of several hundred (in the case of plasma) to thousands (in the case of tissue) of proteins per sample [23,24]. State-of-the-art instrumentation and analytical strategies allow for sufficient sample throughput and accurate quantification, rendering LC–MS/MS a powerful technique for clinical biomarker discovery [25,26,27]. Discovery approaches are often based on tissue analysis and the subsequent transfer of candidates to other assays and easily accessible samples such as plasma. Naturally, many candidates are lost during this attempt and never successfully measured in blood. The analysis of plasma samples circumvents this step, allowing for direct analysis of the sample that is used in routine diagnostics. In addition to this advantage, the enormous dynamic range of the plasma proteome still makes it a challenging kind of sample [28]. Consequently, plasma proteomics studies mostly cover typical plasma proteins, which, nevertheless, might contain valuable information for diagnostics, especially when combined in a multi-biomarker panel [29,30].

We performed a mass-spectrometry-based analysis of plasma and a combined data analysis using univariate statistics and machine learning. We addressed the problem of minimal-invasive differentiation of AC and COPD with the aim of identifying novel biomarker candidates to be implemented in a biomarker panel. To this end, samples from AC patients with and without COPD, COPD patients, and hospital controls (HC) without the target disease were analyzed. COPD and lung cancer are closely linked diseases with a common etiology and similarities in their molecular pathogenesis [31], with COPD being an independent risk factor of lung cancer [7,32]. The effect of inflammatory signaling, for example, has already been shown in COPD [33], but in lung adenocarcinoma, there are also always sites of inflammation [34]. As a consequence, the comparison between AC and HC might result in regulated proteins that might be associated with, e.g., inflammation, but not be specific for AC. In our study, the respective univariate comparisons between diseased groups and HC lead to most significantly differentially abundant proteins, supporting the assumption that many of these are not specific for AC. This highlights the need for the direct comparison of clinically relevant AC and COPD patients to identify proteins that are specific for the respective differentiation. Hence, we focused on the discrimination of AC vs. COPD and the more challenging differentiation of AC with COPD vs. COPD using machine learning.

We constructed the pipeline for model optimization with respect to avoiding overfitting of the trained models. The selected classification algorithms were capable of handling low numbers of observations, and artificial neural networks were not considered, due to their known tendency toward overfitting when used with small datasets. In addition, we selected proteins for model development based on their univariate relevance on the target variable and removed redundant proteins to reduce the overall number of features. Finally, the algorithms were optimized for only ten features and the ten-times-repeated 10-fold cross-validation approach was applied to prevent overoptimistic evaluation of the model’s precision. The comparison of machine learning approaches led to the best results for random forest classification; however, for the comparison of AC with COPD vs. COPD, support vector machines performed comparably well. Both best-performing classifiers yielded cross-validated AUCs over 0.9. To further evaluate our results, we repeated the development of the random forest model for *(A)* AC vs. COPD on 50 randomly selected train-test-set splits with one third of the dataset used for testing. We compared the model characteristics on the test sets to the results on the complete dataset to ensure that the models are not overoptimistic. As expected, the mean AUC of 0.823 in intra-set validation was slightly lower than on the complete dataset but still showed a very good precision. In addition, we compared the most frequently selected features to the results from the complete dataset. Here, four of the five most important features were selected in over 70% of the train-test-splits. For *(B)* AC with COPD vs. COPD, we did not perform the same analysis, because of the limited group size of AC with COPD (n = 21) and the unbalanced nature of the dataset. Although our machine learning approach addressed the problem of overfitting consequently, we must assume that the classifiers are still over-optimistic and should be evaluated with an independent patient cohort before considering them in clinical application. However, the excellent differentiation of clinically very similar patient groups clearly illustrates the great potential of plasma proteomics for biomarker discovery and diagnostics. In addition, the feature importance suggests several proteins to be considered for biomarker panel development.

The proteins that were found to be significantly regulated using univariate statistics or included in the multivariate algorithms were classical plasma proteins. Thus, a pathomechanistic interpretation of the observed differences in abundance is difficult and mostly speculative. Obviously, this was the case for Ig kappa light chain, which was more abundant in AC with or without COPD vs. COPD and found to be a highly informative feature in the classifier for AC vs. COPD. Bottom-up mass spectrometry approaches do not allow routine sequencing of antibody binding sites and the specificity of the regulated Ig chains remains unknown. Free light chains, however, which are synthetized in excess during antibody production, were also shown to be highly bioactive molecules modulating the immune response, e.g., in COPD [35,36]. Although the exact nature of the Ig kappa light chain cannot be clarified based on our data, our findings suggest a robust difference between AC and COPD patients, suggesting its consideration for a biomarker panel. Alpha-1-antichymotrypsin (SERPINA3) belongs to a superfamily of serine proteinase inhibitors. Although its exact biological function is not known, it has been described in association with diverse pathologies, such as acute kidney injury, cardiovascular disease, and (lung) cancer [37,38,39,40]. It was described to promote metastasis and the epithelial–mesenchymal transition in breast cancer [41] and was found to be upregulated in lung cancer in comparison to control patients [42]. In our study, the highest SERPINA3 abundance was found in COPD patients (Supplementary Figure S3), again demonstrating the need for the direct comparison of clinically relevant patient groups. Although SERPINA3 was less abundant compared to COPD, it might be included in a biomarker panel. Its value for differentiation of AC and COPD has been underlined by both univariate statistics and random forest classification. In addition, several other protein candidates should be taken into consideration for the development of a biomarker panel. Apolipoprotein A-IV (APOA4) and Transthyretin (TTR), which were included in random forest classifiers, were both described to be dysregulated in adenocarcinoma before [43]. While APOA4 was less abundant in comparison to nontumorous tissue, TTR was upregulated, which is in concordance with our observations (Supplementary Figure S3). SAA1, which was one of the significantly regulated proteins overlapping between both comparisons of AC with and without COPD vs. COPD, was reported as a biomarker for lung cancer before [44,45,46]. Contrary to most previous reports, SAA1 was significantly downregulated in AC compared to COPD and hospital controls. Notably, most studies reported in the literature used healthy donors as controls, which is not fully comparable with our control group of subjects that were hospitalized under suspicion of lung cancer. Thus, plasma levels of SAA1 should be further studied in clinically relevant patient groups to investigate its true value as a biomarker for lung adenocarcinoma. Notably, intra-set validation supported the value of Ig kappa light chain, SERPINA3, and others, but also highlighted the relevance of the uncharacterized protein C16orf46, which might be further studied in the context of AC and COPD.

## 4. Materials and Methods

### 4.1. Patient Plasma Samples and Clinical Data

Samples and data were collected at the Johanniter-Clinics Bonn and the Malteser Krankenhaus Seliger Gerhard Bonn/Rhein-Sieg. The local ethics committees at the Ruhr-University Bochum, Ärztekammer Nordrhein, and Cologne University approved the study (approval numbers 17-5970, 2017133, 17-162). Written, informed consent was obtained from each patient, and the study protocol conformed to the ethical guidelines of the 1975 Declaration of Helsinki. Peripheral blood was collected in 9 mL S-Monovette EDTA gel tubes (Sarstedt, Nümbrecht, Germany). Within 30 min after blood collection, samples were centrifuged at 2000× *g* for ten minutes at room temperature. Plasma was separated subsequently and frozen immediately at −80 °C until analyses. Samples were measured separately in two batches (n = 63 and n = 133), which were subsequently combined for analysis. In total, plasma samples from 43 AC patients without COPD, 21 AC patients with COPD, 77 COPD patients, and 35 hospital controls (HCs) were analyzed (n = 176, Table 4, Supplementary Table S1). Control subjects were patients with suspicious malignant disease, which was not confirmed subsequently.

### 4.2. Sample Preparation for LC–MS/MS Analysis

Briefly, 1 µL of plasma per sample was mixed with 24 µL of buffer (1% sodium deoxycholate, 10 mM tris (2-carboxyethyl) phosphine, 40 mM chloroacetamide, and 100 mM Tris; pH 8.5) and incubated at 95 °C for 10 min. After cooling down to room temperature, 10 µL of 50 mM ammonium bicarbonate and 10 µL of paramagnetic beads were added according to the SP3 protocol [47]. For protein binding, 140 µL of acetonitrile was added and samples were incubated at room temperature for 10 min. Afterward, the beads were washed twice with 200 µL of 70% ethanol and once with 200 µL of acetonitrile using a magnetic rack. After a short airdrying process, 70 µL of 50 mM ammonium bicarbonate containing trypsin (1:50, *w*/*w*, SERVA Electrophoresis, Heidelberg, Germany) was added to digest the protein overnight at 37 °C. The supernatant was transferred to a glass vial and dried in a vacuum centrifuge. An amount of 100 µL of 0.1% TFA was used to dissolve the peptides and the resulting peptide concentration was about 1 µg/µL.

### 4.3. LC–MS/MS Analysis

The LC–MS/MS analysis was carried out using an Ultimate 3000 RSLCnano liquid chromatography system coupled online to a Q Exactive HF (batch 1) or Q Exactive (batch 2) mass spectrometer (all Thermo Fisher Scientific). Next, 100 ng of plasma peptides per sample were injected for analysis. The peptides were pre-concentrated for 7 min on a trap column (Acclaim^®^ PepMap 100, 75 μm × 2 cm, C18, 5 μm, 100 Å) using 30 μL/min of 0.1% TFA as the loading solvent. Subsequent separation on an analytical column (Acclaim^®^ PepMap RSLC, 75 μm × 50 cm, nano Viper, C18, 5 μm, 100 Å) was carried out using a gradient from 5 to 40% solvent B in solvent A over 98 min (batch 1) or 38 min (batch 2) (solvent A: 0.1% formic acid; solvent B: 0.1% formic acid, 84% acetonitrile). A flow rate of 400 nL/min was used with a column oven temperature of 60 °C. Data-dependent acquisition mode was used. Full scans were acquired in the Orbitrap analyzer (mass range: 350–1400 m/z, resolution: 60,000). The Fourier Transform Mass Spectrometry full-scan Automatic Gain Control target was set to 3 × 10^6^ with a maximum injection time of 80 ms. The number of micro-scans was set to 1. The 10 most abundant ions of a spectrum acquired at the MS1 level were fragmented using HCD (higher-energy collisional dissociation) with a normalized collision energy of 30% and an isolation width of 2 m/z. Fragment mass spectra were acquired in the orbitrap with a maximum injection time of 100 ms. Samples were measured in a random sequence. The mass spectrometry proteomics data have been deposited to the ProteomeXchange Consortium via the PRIDE partner repository with the dataset identifier PXD035120 and 10.6019/PXD035120”.

### 4.4. Protein Identification and Quantification

Protein identification and label-free quantification were performed using MaxQuant (version 2.0.1.0; MPI of Biochemistry, Martinsried, Germany) separately for both analyzed batches. Unless specifically mentioned, the MaxQuant default parameters were used. Spectra were searched against the Uniprot/Swissprot database version 2021_03 restricted to *Homo sapiens* (20.362 entries) and Biognosys iRT peptides (1 entry). Variable modifications of methionine (oxidation) and protein N-termini (acetylation) were considered as well as fixed modification of cysteine (carbamidomethyl). Modified peptides were considered for quantification and the match between runs function was enabled. The false discovery rate (FDR) was set to 0.01 for peptide-spectrum matches (PSMs) and proteins. Protein groups were used for further processing and reverse hits, proteins were identified by site, and identified iRT peptides were omitted. Potential contaminants were manually reviewed to avoid exclusion of typical plasma proteins.

### 4.5. Batch Normalization

LFQ intensities were normalized to reduce technical variation and the batch effects between the two batches to allow analyzing them together. First, each batch was normalized separately using the LOESS normalization method [48] using the R package limma (version 3.44.3) [49] on the log-transformed protein intensities. For each protein, a linear regression model was calculated by using the batch number as a categorical independent variable. The obtained coefficients were an estimation of the underlying batch effect, which were subsequently subtracted from the protein intensities. This normalization procedure reduces the batch differences for better comparability of the samples. As the LC gradient was adapted for the second batch due to the higher sample amount and a different instrument, 20 samples of the first batch were also measured in the second batch and taken as a quality control for normalization. The quality of the normalization was evaluated using boxplots, PCA (principal component analysis), and MA plots (Figure 2, Supplementary Figures S1 and S2).

### 4.6. Statistical Analysis

The statistical analysis was conducted using R version 4.0.3 (R Core Team 2020, Vienna, Austria). For this, the intensities of the doubly measured samples were averaged after the batch normalization. Protein groups quantified in a minimum of five patients in the compared patient groups were considered for testing. In order to identify significantly different protein groups between the experimental groups, normalized intensities were analyzed by application of a Welch-ANOVA (R package car version 3.0-10). This was followed by single Welch tests for each protein as a post hoc method in all possible pairwise group comparisons. Ratios of means (RoMs) between groups were determined on the delogarithmized intensities. The FDR was controlled by adjusting ANOVA p-values using the method of Benjamini and Hochberg [50]. Post hoc *p*-values were adjusted by the method of Bonferroni–Holm [51] for each protein separately. Proteins were considered significant with *p*_FDR_-values ≤ 0.05 (from ANOVA and post hoc test) and an absolute RoM ≥ 1.5. For the comparison of AC vs. COPD, a separate Welch test was used and corrected according to Benjamini–Hochberg.

### 4.7. Machine Learning

The normalized protein intensities were used to develop classification models. For the two research questions, separation of *(1)* AC vs. COPD and *(2)* AC with COPD vs. COPD, the same pipeline for model optimization, consisting of feature selection and model optimization, was used (Figure 1). Proteins with more than 30% missing values or a variance near zero across all samples were omitted. In addition, redundant proteins were identified according to Spearman’s rank correlation coefficient >0.7 and removed from analysis. For feature selection, proteins were selected according to their influence on the respective target variable. Therefore, Kruskal–Wallis and Levene tests were calculated. *p*-value thresholds of 0.05, 0.1, 0.2, 0.3, and 0.5 were applied to optimize this feature selection. In addition, the performance without feature selection by statistical tests was assessed. For model optimization, logistic regression, linear discriminant analysis (LDA), and support vector machines (SVMs) with linear as well as polynomial kernel and random forest were applied and compared. We optimized the random forest and SVM models for the best subset of ten proteins from the selected features to reduce overfitting. For LDA and logistic regression, the best subset was determined by the recursive feature selection procedure. The models were trained with the ten-times-repeated 10-fold cross-validation approach using the area under the precision recall curve (PRAUC) as the optimization criterion, which was chosen to cope with the unbalanced datasets. The models with the highest AUC (area under the ROC curve) and PRAUC were analyzed according to permutation feature importance (https://doi.org/10.48550/arXiv.2006.04628 (accessed on 9 February 2022)). Permutation feature importance calculates the increase in the root-mean-square error (RSME) of the prediction model when a single feature value is randomly shuffled. The increase in RSME is indicative of how much the model depends on the feature. In addition, we repeated the development of the final model on 50 randomly selected train-test-splits with 2/3 of the dataset selected for model development and 1/3 as the test set for validation. We compared the model characteristics on the test sets to the results on the complete dataset and evaluated the number of repetitions in which a feature was selected by the final model.

## 5. Conclusions

A realistic protein biomarker panel in clinical use needs to be limited to a manageable number of proteins, which, at least in the nearer future, will be measured by multiplex ELISA or comparable techniques. These requirements limit the variety of biomarker candidates for assay development. Therefore, the considered proteins need to address a specific clinical problem, which was analyzed by use of the appropriate clinical groups. Here, we describe a panel of proteins that were differentially abundant between patients with AC and COPD, while taking into consideration that many AC patients also suffer from underlying COPD. Addressing this problem represents the first and urgently necessary step in discovery of reliable markers that distinguish between early AC, COPD, and their overlaps. The combination of univariate statistics and machine learning adds complementary information and, thus, provides insights that would not be available with either approach alone: Classification algorithms strive for the highest diagnostic precision while permutation feature importance or univariate statistics allow identification of the most influential proteins. Those proteins that were identified in this study might serve as starting points for development of a biomarker panel, which addresses the heterogeneity of AC and COPD, ideally in an early detection setting.

## Figures and Tables

**Table 1:** Significantly differentially abundant protein groups.

Comparison (Condition A vs. Condition B)	Protein Groups Considered for Statistical Testing ^1^	Significantly Differentially Abundant Protein Groups ^2^	Higher Abundance in Condition A	Higher Abundance in Condition B
AC with COPD vs. COPD	325	**11**	3	8
AC w/o COPD vs. COPD	349	**11**	6	5
AC w/o COPD vs. AC with COPD	324	**3**	3	0
AC with COPD vs. Control	271	**39**	14	25
AC w/o COPD vs. Control	278	**26**	9	17
COPD vs. Control	283	**31**	14	18

^1^ Protein groups were filtered for a minimum of five valid quantifications per patient group. ^2^ Significance filter criteria: ANOVA *p*_FDR_ and post hoc *p*_FDR_-values ≤ 0.05; absolute RoM ≥ 1.5.

**Table 2:** Characteristics of random forest classifiers with the highest AUCs *.

*(1)* AC vs. COPD ^1^		*(2)* AC with COPD vs. COPD ^2^	
**AUC**	**0.935**	**AUC**	0.916
**PRAUC**	0.928	**PRAUC**	0.882
**Accuracy**	0.865	**Accuracy**	0.873
**Sensitivity ^3^**	0.848	**Sensitivity ^5^**	0.570
**Specificity ^4^**	0.879	**Specificity ^4^**	0.965

* Ten-times-repeated 10-fold cross-validated. ^1^ For feature selection, a *p*-value threshold of 0.2 was used. ^2^ No *p*-value threshold was applied for feature selection. ^3^ Sensitivity corresponds to true classification of AC. ^4^ Specificity corresponds to true classification of COPD. ^5^ Sensitivity corresponds to true classification of AC with COPD.

**Table 3:** Metrics for train-test-split validation for the comparison AC vs. COPD.

		Minimum ^1^	Mean ^1^	Maximum ^1^
**AUC**	**Train set ^2^**	0.85	0.901	0.965
	**Test set ^3^**	0.667	0.823	0.936
**PRAUC**	**Train set**	0.763	0.864	0.968
	**Test set**	0.554	0.766	0.931
**Accuracy**	**Train set**	0.76	0.831	0.91
	**Test set**	0.65	0.753	0.85
**Sensitivity**	**Train set**	0.726	0.815	0.905
	**Test set**	0.5	0.763	1
**Specificity**	**Train set**	0.759	0.844	0.941
	**Test set**	0.455	0.745	1

^1^ Model metrics represent 50 repetitions of random train-test-splits. ^2^ Model built on train set with ten-times-repeated 10-fold cross-validation. ^3^ Model validated on test set representing 1/3 of the whole dataset.

**Table 4:** Composition of the analyzed patient cohorts.

Group		Description	Mean Age (Years)	Sex	Smoking Behavior
AC * **(n = 64)**	AC w/o COPD (n = 43)	AC-patients without diagnosed COPD	67.17 ± 9.43, min. 41, max. 85	25 female, 18 male	20 smokers, 10 ex-smokers, 13 never-smokers
	AC with COPD (n = 21)	AC-patients with diagnosed COPD	64.48 ± 8.89, min. 52, max. 84	12 female, 9 male	11 smokers, 8 ex-smokers, 2 never-smokers
COPD ^§^ (n = 77)		COPD-patients without AC	68.61 ± 10.43, min. 38, max. 87	36 female, 41 male	36 smokers, 33 ex-smokers, 6 never-smokers, 2 NA
HC (n = 35)		Hospital controls	65.34 ± 12.40 min. 41, max. 82	16 female, 19 male	14 smokers, 13 ex-smokers, 8 never-smokers

* Lung adenocarcinoma. ^§^ Chronic obstructive pulmonary disease.

## Data Availability

The mass spectrometry proteomics data have been deposited to the ProteomeXchange Consortium via the PRIDE partner repository with the dataset identifier PXD035120 and 10.6019/PXD035120.

## Supplementary Materials

The following supporting information can be downloaded at: https://www.mdpi.com/article/10.3390/ijms231911242/s1.
