# Supplementary material for: Plasma Proteomics Enable Differentiation of Lung Adenocarcinoma from Chronic Obstructive Pulmonary Disease (COPD)
Source: Int J Mol Sci. 2022 Sep 24;23(19):11242. doi: 10.3390/ijms231911242 (PMC9569607; doi:10.3390/ijms231911242)
Supplement: Supplementary file 1 [file ijms-23-11242-s001.zip › ijms-1861072 -Supplementary Table S1- Description of the Analyzed Patient Cohorts.pdf]

Supplementary Table S1 - Composition of the analyzed patient cohorts

[1] Lung adenocarcinoma

[2] Chronic obstructive pulmonary disea

	Sample ID	MS ID Batch1	MS ID Batch2	Patient ID
**AC[1] w/o COPD[2]**	A22	QExHF12040		L20004
	A23	QExHF12046		L20007
	A24	QExHF12052		L20012
	A25	QExHF12058	QEXI28055	L20013
	A26	QExHF12064		L20014
	A27	QExHF12074		L20015
	A30	QExHF12092		L20043
	A31	QExHF12098		L20048
	A32	QExHF12104		L20051
	A33	QExHF12114		L20053
	A34	QExHF12120		L20058
	A35	QExHF12126		L20076
	A38	QExHF12144	QEXI28264	L20085
	A39	QExHF12156	QEXI28270	L20086
	A41	QExHF12168	QEXI28282	L20093
	A42	QExHF12174	QEXI28288	L20095
	A45	QExHF12196	QEXI28306	L20104
	A47	QExHF12208	QEXI27877	L20136
	A48	QExHF12214	QEXI27883	L20140
	A49	QExHF12236	QEXI27889	L20148
	A51	QExHF12248	QEXI27901	L20166
	A53		QEXI27913	L20179
	A54		QEXI27923	L20181
	A55		QEXI27929	L20186
	A56		QEXI27935	L20190
	A61		QEXI27965	L20216
	A63		QEXI27983	L20220
	A65		QEXI27995	L20233
	A66		QEXI28001	L20238
	A67		QEXI28031	L20239
	A68		QEXI28037	L20249
	A70		QEXI28049	L20256
	A72		QEXI28061	L20271
	A75		QEXI28081	L20280
	A76		QEXI28089	L20287
	A77		QEXI28344	L20288
	A79		QEXI28107	L20290
	A80		QEXI28113	L20294
	A82		QEXI28129	L20319
	A84		QEXI28146	L20332

	A85		QEXI28152	L20337
	A87		QEXI28164	L20345
	A88		QEXI28170	L20349
	**n = 43**			**mean**
				**SD**
				**min**
				**max**
				**male**
				**female**
				**smoker**
				**never-smoker**
				**ex-smoker**
**AC[1] with COPD[2]**	AO21	QExHF12034		L20003
	AO29	QExHF12086		L20040
	AO36	QExHF12132		L20078
	AO37	QExHF12138	QEXI28258	L20083
	AO40	QExHF12162	QEXI28276	L20087
	AO43	QExHF12180	QEXI28294	L20097
	AO44	QExHF12186	QEXI28300	L20101
	AO46	QExHF12202	QEXI27871	L20110
	AO50	QExHF12242	QEXI27895	L20163
	AO52	QExHF12254	QEXI27907	L20178
	AO57		QEXI27941	L20205
	AO58		QEXI27947	L20206
	AO59		QEXI27953	L20209
	AO60		QEXI27959	L20213
	AO62		QEXI27977	L20219
	AO64		QEXI27989	L20222
	AO69		QEXI28043	L20252
	AO73		QEXI28067	L20273
	AO74		QEXI28075	L20274
	AO78		QEXI28101	L20289
	AO83		QEXI28138	L20320
	**n = 21**			**mean**
				**SD**
				**min**
				**max**
				**male**
				**female**
				**smoker**
				**never-smoker**
				**ex-smoker**
**Control**	C01		QEXI27911	L20061
	C03		QEXI27927	L20077

	C04	QEXI27933	L20081
	C05	QEXI27939	L20091
	C06	QEXI27945	L20098
	C07	QEXI27951	L20102
	C08	QEXI27957	L20109
	C09	QEXI27963	L20116
	C10	QEXI27969	L20118
	C11	QEXI27981	L20119
	C12	QEXI27987	L20131
	C13	QEXI27993	L20133
	C14	QEXI27999	L20134
	C15	QEXI28005	L20138
	C16	QEXI28009	L20142
	C18	QEXI28017	L20151
	C19	QEXI28021	L20153
	C20	QEXI28127	L20158
	C21	QEXI28136	L20160
	C23	QEXI28398	L20169
	C24	QEXI28404	L20180
	C25	QEXI28410	L20185
	C26	QEXI28416	L20187
	C27	QEXI28422	L20188
	C29	QEXI28438	L20196
	C30	QEXI28444	L20199
	C31	QEXI28450	L20207
	C32	QEXI28456	L20211
	C33	QEXI28462	L20214
	C34	QEXI28468	L20230
	C35	QEXI28231	L20241
	C36	QEXI28235	L20242
	C37	QEXI28239	L20247
	C38	QEXI28243	L20248
	C39	QEXI28474	L20250
	**n=35**		**mean**
			**SD**
			**min**
			**max**
			**male**
			**female**
			**smoker**
			**never-smoker**
			**ex-smoker**
**COPD**[2]	O1	QExHF12036	L20002
	O2	QExHF12042	L20011
	O3	QExHF12048	L20020

O4	QExHF12054		L20021
O5	QExHF12060		L20022
O6	QExHF12066		L20023
O7	QExHF12076		L20024
O8	QExHF12082		L20026
O9	QExHF12088		L20027
O10	QExHF12094		L20028
O11	QExHF12100		L20029
O12	QExHF12106		L20033
O13	QExHF12116		L20034
O14	QExHF12122		L20049
O15	QExHF12128		L20050
O16	QExHF12134		L20055
O17	QExHF12140		L20056
O18	QExHF12146		L20064
O19	QExHF12158		L20065
O20	QExHF12164		L20066
O21	QExHF12170		L20070
O22	QExHF12176		L20089
O23	QExHF12182		L20090
O24	QExHF12188		L20106
O25	QExHF12198		L20107
O26	QExHF12204		L20141
O27	QExHF12210		L20155
O28	QExHF12216		L20195
O29	QExHF12238		L20225
O30	QExHF12244	QEXI28260	L20226
O31	QExHF12250	QEXI28266	L20227
O32		QEXI28272	L20231
O33		QEXI28278	L20232
O34		QEXI28284	L20235
O35		QEXI28290	L20236
O36		QEXI28296	L20243
O37		QEXI28302	L20254
O38		QEXI27867	L20255
O39		QEXI27873	L20259
O40		QEXI27879	L20261
O41		QEXI27885	L20262
O42		QEXI27891	L20267
O43		QEXI27897	L20276
O44		QEXI27903	L20278
O45		QEXI27909	L20281
O46		QEXI27915	L20282
O47		QEXI27925	L20025
O65		QEXI28025	L20297
O67		QEXI28039	L20300

O68	QEXI28045	L20302
O71	QEXI28063	L20307
O72	QEXI28069	L20308
O73	QEXI28077	L20309
O74	QEXI28085	L20311
O75	QEXI28340	L20313
O76	QEXI28123	L20316
O77	QEXI28103	L20317
O78	QEXI28109	L20321
O79	QEXI28115	L20324
O80	QEXI28125	L20326
O81	QEXI28131	L20330
O82	QEXI28390	L20331
O83	QEXI28396	L20335
O84	QEXI28402	L20336
O85	QEXI28160	L20339
O86	QEXI28414	L20341
O87	QEXI28420	L20342
O88	QEXI28426	L20344
O91	QEXI28448	L20355
O92	QEXI28454	L20356
O93	QEXI28460	L20357
O96	QEXI28233	L20365
O97	QEXI28237	L20366
O98	QEXI28241	L20367
O99	QEXI28245	L20370
O100	QEXI28476	L20372
O101	QEXI28478	L20375
**n = 77**		**mean**
		**SD**
		**min**
		**max**
		**male**
		**female**
		**smoker**
		**never-smoker**
		**ex-smoker**
		NA

Age	Sex	Smoking status	COPD[2]
56	male	smoker	no
41	male	smoker	no
74	female	never-smoker	no
67	male	smoker	no
69	male	never-smoker	no
64	male	smoker	no
68	male	smoker	no
77	female	never-smoker	no
64	female	smoker	no
74	male	never-smoker	no
73	male	smoker	no
61	female	never-smoker	no
50	male	smoker	no
70	male	ex-smoker	no
85	female	never-smoker	no
73	male	smoker	no
77	male	smoker	no
68	male	smoker	no
53	male	never-smoker	no
60	female	smoker	no
74	female	never-smoker	no
76	female	never-smoker	no
68	female	smoker	no
63	female	smoker	no
76	female	never-smoker	no
76	female	ex-smoker	no
78	female	ex-smoker	no
82	male	ex-smoker	no
73	female	never-smoker	no
62	female	smoker	no
71	female	never-smoker	no
70	female	smoker	no
71	male	ex-smoker	no
47	female	ex-smoker	no
59	female	ex-smoker	no
68	male	smoker	no
63	female	smoker	no
65	female	smoker	no
63	female	ex-smoker	no
54	female	ex-smoker	no

66	male	smoker	no
62	female	never-smoker	no
77	female	ex-smoker	no
**67.16**			
**9.32**			
**41**			
**85**			
**18**			
**25**			
**20**			
**13**			
**10**			
72	female	smoker	yes
56	female	smoker	yes
65	male	ex-smoker	yes
75	female	ex-smoker	yes
55	male	smoker	yes
53	male	ex-smoker	yes
61	male	smoker	yes
69	male	ex-smoker	yes
68	male	ex-smoker	yes
63	female	never-smoker	yes
62	female	smoker	yes
69	female	ex-smoker	yes
60	male	smoker	yes
75	female	smoker	yes
84	female	never-smoker	yes
53	female	smoker	yes
68	female	smoker	yes
77	male	ex-smoker	yes
52	male	smoker	yes
57	female	ex-smoker	yes
60	female	smoker	yes
**64.48**			
**8.89**			
**52**			
**84**			
**9**			
**12**			
**11**			
**2**			
**8**			
55	female	ex-smoker	no
80	male	ex-smoker	no

57	male	smoker	no
52	female	never-smoker	no
52	male	ex-smoker	no
73	male	ex-smoker	no
76	female	never-smoker	no
69	male	smoker	no
59	male	smoker	no
77	female	ex-smoker	no
66	male	smoker	no
51	female	never-smoker	no
41	female	never-smoker	no
82	male	smoker	no
66	male	smoker	no
74	female	ex-smoker	no
75	male	smoker	no
60	male	ex-smoker	no
43	female	smoker	no
51	female	never-smoker	no
58	male	smoker	no
66	female	never-smoker	no
59	female	never-smoker	no
81	female	smoker	no
59	male	smoker	no
77	male	ex-smoker	no
64	female	ex-smoker	no
58	female	smoker	no
82	male	ex-smoker	no
81	male	ex-smoker	no
73	female	smoker	no
41	male	ex-smoker	no
80	female	ex-smoker	no
72	male	smoker	no
77	male	never-smoker	no
**65.34**			
**12.40**			
**41.00**			
**82.00**			
**19**			
**16**			
**14**			
**8**			
**13**			
55	male	smoker	yes
76	male	smoker	yes
47	female	ex-smoker	yes

77	female	smoker	yes
78	female	ex-smoker	yes
66	female	smoker	yes
77	male	smoker	yes
59	female	smoker	yes
66	male	smoker	yes
87	male	ex-smoker	yes
67	female	never-smoker	yes
58	male	ex-smoker	yes
56	male	smoker	yes
63	male	smoker	yes
75	female	ex-smoker	yes
74	male	ex-smoker	yes
69	male	ex-smoker	yes
62	male	ex-smoker	yes
70	female	ex-smoker	yes
46	female	smoker	yes
73	male	smoker	yes
69	female	ex-smoker	yes
74	female	NA	yes
55	male	smoker	yes
59	male	ex-smoker	yes
82	female	never-smoker	yes
64	male	smoker	yes
69	female	ex-smoker	yes
56	female	ex-smoker	yes
65	male	ex-smoker	yes
82	female	never-smoker	yes
82	male	never-smoker	yes
83	male	ex-smoker	yes
70	male	ex-smoker	yes
81	male	never-smoker	yes
78	female	ex-smoker	yes
65	male	smoker	yes
52	male	smoker	yes
64	female	ex-smoker	yes
58	male	ex-smoker	yes
65	male	smoker	yes
82	female	ex-smoker	yes
38	female	smoker	yes
62	male	ex-smoker	yes
71	female	ex-smoker	yes
52	male	smoker	yes
78	female	smoker	yes
81	male	ex-smoker	yes
59	male	smoker	yes

80	male	ex-smoker	yes
64	female	smoker	yes
81	male	NA	yes
72	female	smoker	yes
64	male	smoker	yes
61	male	smoker	yes
71	female	ex-smoker	yes
84	male	ex-smoker	yes
58	female	smoker	yes
80	female	never-smoker	yes
76	male	smoker	yes
80	female	smoker	yes
77	male	smoker	yes
76	male	ex-smoker	yes
80	female	smoker	yes
60	male	smoker	yes
79	male	ex-smoker	yes
83	female	ex-smoker	yes
73	female	smoker	yes
68	female	ex-smoker	yes
72	male	smoker	yes
65	male	smoker	yes
64	female	ex-smoker	yes
75	female	ex-smoker	yes
52	female	smoker	yes
57	male	smoker	yes
65	female	smoker	yes
69	female	ex-smoker	yes
**68.61**			
**10.43**			
**38**			
**87**			
**41**			
**36**			
**36**			
**6**			
**33**			
**2**			
