# Supplementary material for: Plasma Proteomics Enable Differentiation of Lung Adenocarcinoma from Chronic Obstructive Pulmonary Disease (COPD)
Source: Int J Mol Sci. 2022 Sep 24;23(19):11242. doi: 10.3390/ijms231911242 (PMC9569607; doi:10.3390/ijms231911242)
Supplement: Supplementary file 1 [file ijms-23-11242-s001.zip › ijms-1861072-Supplementary Information.pdf]

Plasma proteomics enable differentiation of lung adenocarcinoma from chronic obstructive pulmonary disease (COPD)

Thilo Bracht[1, 2], Daniel Kleefisch[2, 3], Karin Schork[2, 3], Kathrin E. Witzke[2, 3], Weiqiang Chen[2], Malte Bayer[1, 2], Jan Hovanec[4], Georg Johnen[4], Swetlana Meier[4], Yon-Dschun Ko[5], Thomas Behrens[4], Thomas Brüning[4], Jana Fassunke[6], Reinhard Buettner[6], Julian Uszkoreit[2, 3], Michael Adamzik[1], Martin Eisenacher[2,3*], and Barbara Sitek[1,2*]

Supplementary Information

Supplementary Table S4. Significantly differentially abundant proteins (ANOVA)
Filter criteria: ANOVA p_{FDR}-value ≤ 0.05 (Benjamini-Hochberg-corrected), post hoc test p-value ≤ 0.05 (Welch test, Bonferroni-Holm-corrected), absolute ratio of means ≥ 1.5.

(A) Adeno with COPD vs. COPD

UniProt ID	Gene Name	Protein Name	ANOVA p_{FDR}-value	Post hoc p-value	Ratio of means*
P01011	SERPINA3	Alpha-1-antichymotrypsin	1.05E-10	1.30E-05	0.64
P0DOX7	*NaN*	Immunoglobulin kappa light chain	9.63E-06	7.34E-06	1.51
P0DJI8	SAA1	Serum amyloid A-1 protein	1.21E-03	3.06E-03	0.10
P02763	ORM1	Alpha-1-acid glycoprotein 1	2.17E-03	3.60E-02	0.67
A0A087WSY6	IGKV3D-15	Immunoglobulin kappa variable 3D-15	2.17E-03	5.59E-03	0.63
A0A075B6S5	IGKV1-27	Immunoglobulin kappa variable 1-27	3.08E-03	1.95E-04	1.66
P10643	C7	Complement component C7	7.63E-03	2.67E-03	0.66
P01817	IGHV2-5	Ig heavy chain V-II region MCE	1.19E-02	2.46E-03	3.30
O00391	QSOX1	Sulfhydryl oxidase 1	1.54E-02	1.73E-02	0.53
Q08380	LGALS3BP	Galectin-3-binding protein	2.37E-02	2.44E-02	0.60
P68871	HBB	Hemoglobin subunit beta;	2.51E-02	2.34E-02	0.58

(B) Adeno w/o COPD vs. COPD

UniProt ID	Gene Name	Protein Name	ANOVA p_{FDR}-value	Post hoc p-value	Ratio of means*
P0DOX7	*NaN*	Immunoglobulin kappa light chain	9.63E-06	1.04E-06	1.51
P11226	MBL2	Mannose-binding protein C	8.78E-05	2.40E-02	0.54
P0DJI8	SAA1	Serum amyloid A-1 protein	1.21E-03	2.94E-03	0.15
Q12805	EFEMP1	EGF-containing fibulin-like extracellular matrix protein 1	2.17E-03	2.18E-02	0.62
P04003	C4BPA	C4b-binding protein alpha chain	3.79E-03	2.37E-04	1.51
Q92686	NRGN	Neurogranin;NEUG(55-78)	9.05E-03	2.48E-03	1.67
A0A0B4J2H0	IGHV1-69D	Immunoglobulin heavy variable 1-69D	1.31E-02	3.74E-02	1.59
O00391	QSOX1	Sulfhydryl oxidase 1	1.54E-02	7.88E-03	0.58
P04275	VWF	von Willebrand factor;von Willebrand antigen 2	2.11E-02	2.36E-03	0.40
Q9GZQ6	NPFFR1	Neuropeptide FF receptor 1	3.68E-02	7.53E-03	1.83
A0A0C4DH43 P01814	IGHV2-70D	Immunoglobulin heavy variable 2-70D	4.94E-02	1.24E-02	2.05

(C) Adeno w/o COPD vs. Adeno with COPD

UniProt ID	Gene Name	Protein Name	ANOVA p_{FDR}-value	Post hoc p-value	Ratio of means*
A0A087WSY6	IGKV3D-15	Immunoglobulin kappa variable 3D-15	2.17E-03	4.97E-02	1.66
Q92686	NRGN	Neurogranin;NEUG(55-78)	9.05E-03	2.20E-02	1.60
A0A0B4J2H0	IGHV1-69D	Immunoglobulin heavy variable 1-69D	1.31E-02	9.69E-03	2.46

(D) Adeno with COPD vs. Control

UniProt ID	Gene Name	Protein Name	ANOVA p_{FDR}-value	Post hoc *p*-value	Ratio of means*
P63104	YWHAZ	14-3-3 protein zeta/delta	2.10E-04	3.59E-05	2.14
Q01518	CAP1	Adenylyl cyclase-associated protein 1	1.98E-03	1.73E-03	5.59
P02763	ORM1	Alpha-1-acid glycoprotein 1	2.17E-03	6.61E-04	0.45
P04114	APOB	Apolipoprotein B-100	1.77E-07	1.13E-08	1.67
Q13790	APOF	Apolipoprotein F	3.97E-02	3.55E-02	1.50
O95445	APOM	Apolipoprotein M	1.69E-02	1.88E-02	0.50
P02749	APOH	Beta-2-glycoprotein 1	9.01E-03	2.95E-02	0.19
P05160	F13B	Coagulation factor XIII B chain	4.94E-05	3.22E-04	0.31
Q12805	EFEMP1	EGF-containing fibulin-like extracellular matrix protein 1	2.17E-03	3.15E-02	0.64
Q86UX7	FERMT3	Fermitin family homolog 3	3.09E-02	4.69E-03	1.71
Q9UGM5	FETUB	Fetuin-B	2.31E-02	9.13E-03	1.56
P68871	HBB	Hemoglobin subunit beta	2.51E-02	4.03E-02	0.50
P01876	IGHA1	Ig alpha-1 chain C region	4.58E-02	1.44E-02	0.61
P01860	IGHG3	Ig gamma-3 chain C region	8.24E-03	1.76E-02	0.42
P06312	IGKV4-1	Ig kappa chain V-IV region	1.22E-04	2.32E-02	0.55
P01700	IGLV1-47	Ig lambda chain V-I region HA	4.33E-06	1.47E-06	2.33
P80748	IGLV3-21	Ig lambda chain V-III region LOI	2.26E-03	1.96E-02	1.56
A0A0C4DH31	IGHV1-18	Immunoglobulin heavy variable 1-18	2.48E-02	4.84E-02	0.49
P01780	IGHV3-7	Immunoglobulin heavy variable 3-7	4.92E-02	2.98E-02	1.65
A0A0B4J1U7	IGHV6-1	Immunoglobulin heavy variable 6-1	3.97E-02	3.17E-02	0.54
A0A087WSY6	IGKV3D-15	Immunoglobulin kappa variable 3D-15	2.17E-03	2.99E-04	0.55
A0A0C4DH25	IGKV3D-20	Immunoglobulin kappa variable 3D-20	8.22E-04	8.85E-05	0.59
A0A0B4J1U3	IGLV1-36	Immunoglobulin lambda variable 1-36	4.24E-03	1.69E-03	0.28
P01703	IGLV1-40	Immunoglobulin lambda variable 1-40	1.22E-04	6.20E-04	0.37
P11226	MBL2	Mannose-binding protein C	8.78E-05	2.40E-02	0.52
P08571	CD14	Monocyte differentiation antigen CD14	8.73E-03	8.08E-03	0.64
P02776 P10720	PF4; PF4V1	Platelet factor 4	7.63E-03	1.22E-02	0.48
P08567	PLEK	Pleckstrin	1.19E-03	4.90E-02	1.73
P00734	F2	Prothrombin	1.61E-02	2.95E-03	0.55
P02753	RBP4	Retinol-binding protein 4	1.18E-05	4.36E-05	0.25
P02787	TF	Serotransferrin	3.44E-03	2.40E-03	0.59
P0DJI8	SAA1	Serum amyloid A-1 protein	1.21E-03	1.24E-02	0.07

P05543	SERPINA7	Thyroxine-binding globulin	3.44E-03	3.05E-04	1.63
P02766	TTR	Transthyretin	1.07E-02	3.00E-03	1.65
P25311	AZGP1	Zinc-alpha-2-glycoprotein	3.97E-02	4.67E-02	0.31
P0DOY3	IGLC3	Immunoglobulin lambda constant 3	1.83E-07	5.68E-07	0.57
P05452	CLEC3B	Tetranectin	4.32E-05	9.78E-06	2.19
A0A075B6H9	IGLV4-69	Immunoglobulin lambda variable 4-69	4.92E-03	8.16E-04	2.30
A0A075B6P5 P01615 A0A087WW87 P01614	IGKV2D-28 IGKV2-40	Ig kappa chain V-II region FR	8.64E-03	1.71E-03	0.52

(E) Adeno w/o COPD vs. Control

**UniProt ID**	**Gene Name**	**Protein Name**	**ANOVA p_{FDR}-value**	**Post hoc *p*-value**	**Ratio of means***
P0DOY3	IGLC3	Immunoglobulin lambda constant 3	1.83E-07	9.79E-06	0.56
P01700	IGLV1-47	Ig lambda chain V-I region HA	4.33E-06	2.33E-06	2.02
P02753	RBP4	Retinol-binding protein 4	1.18E-05	1.98E-05	0.26
P05452	CLEC3B	Tetranectin	4.32E-05	2.73E-03	1.88
P05160	F13B	Coagulation factor XIII B chain	4.94E-05	2.17E-03	0.43
P11226	MBL2	Mannose-binding protein C	8.78E-05	3.40E-05	0.43
P06312	IGKV4-1	Ig kappa chain V-IV region	1.22E-04	1.05E-03	0.61
P01703	IGLV1-40	Immunoglobulin lambda variable 1-40	1.22E-04	6.97E-04	0.41
P63104	YWHAZ	14-3-3 protein zeta/delta	2.10E-04	2.49E-04	2.19
P08567	PLEK	Pleckstrin	1.19E-03	1.59E-03	2.35
P0DJI8	SAA1	Serum amyloid A-1 protein	1.21E-03	2.33E-02	0.11
Q01518	CAP1	Adenylyl cyclase-associated protein 1	1.98E-03	4.42E-03	4.88
P02763	ORM1	Alpha-1-acid glycoprotein 1	2.17E-03	2.78E-03	0.54
Q12805	EFEMP1	EGF-containing fibulin-like extracellular matrix protein 1	2.17E-03	7.12E-04	0.55
P80748	IGLV3-21	Ig lambda chain V-III region LOI	2.26E-03	1.43E-04	1.93
P02787	TF	Serotransferrin	3.44E-03	2.40E-03	0.62
A0A0B4J1U3	IGLV1-36	Immunoglobulin lambda variable 1-36	4.24E-03	2.51E-03	0.35
P05106	ITGB3	Integrin beta-3	7.04E-03	6.09E-04	2.03
P02776; P10720	PF4; PF4V1	Platelet factor 4	7.63E-03	1.09E-02	0.55
P01860	IGHG3	Ig gamma-3 chain C region	8.24E-03	3.46E-02	0.49
A0A075B6P5 P01615 A0A087WW87 P01614	IGKV2D-28 IGKV2-40	Ig kappa chain V-II region FR	8.64E-03	2.71E-02	0.65

P02749	APOH	Beta-2-glycoprotein 1	9.01E-03	3.55E-02	0.21
P00734	F2	Prothrombin	1.61E-02	1.93E-02	0.64
P01717	IGLV3-25	Immunoglobulin lambda variable 3-25	1.69E-02	1.08E-02	1.76
O95445	APOM	Apolipoprotein M	1.69E-02	3.73E-03	0.48
Q13790	APOF	Apolipoprotein F	3.97E-02	1.18E-02	1.62

(F) COPD vs. Control

**UniProt ID**	**Gene Name**	**Protein Name**	**ANOVA p_{FDR}-value**	**Post hoc *p*-value**	**Ratio of means***
P01011	SERPINA3	Alpha-1-antichymotrypsin	1.05E-10	3.46E-09	1.95
P0DOY3	IGLC3	Immunoglobulin lambda constant 3	1.83E-07	9.54E-07	0.58
P01700	IGLV1-47	Ig lambda chain V-I region HA	4.33E-06	2.61E-04	1.78
P0DOX7	*NaN*	Immunoglobulin kappa light chain	9.63E-06	3.92E-03	0.66
P02753	RBP4	Retinol-binding protein 4	1.18E-05	1.24E-06	0.25
P05160	F13B	Coagulation factor XIII B chain	4.94E-05	5.18E-06	0.34
P01703	IGLV1-40	Immunoglobulin lambda variable 1-40	1.22E-04	6.37E-06	0.34
P63104	YWHAZ	14-3-3 protein zeta/delta	2.10E-04	3.73E-04	1.96
P12259	F5	Coagulation factor V	4.99E-04	8.12E-05	1.72
P08567	PLEK	Pleckstrin	1.19E-03	1.35E-04	2.48
Q01518	CAP1	Adenylyl cyclase-associated protein 1	1.98E-03	7.32E-03	3.71
P80748	IGLV3-21	Ig lambda chain V-III region LOI	2.26E-03	1.15E-02	1.70
P02787	TF	Serotransferrin	3.44E-03	3.72E-04	0.59
P05543	SERPINA7	Thyroxine-binding globulin	3.44E-03	1.91E-03	1.53
A0A0B4J1U3	IGLV1-36	Immunoglobulin lambda variable 1-36	4.24E-03	2.51E-03	0.36
A0A075B6H9	IGLV4-69	Immunoglobulin lambda variable 4-69	4.92E-03	1.97E-02	1.69
P02776 P10720	PF4; PF4V1	Platelet factor 4	7.63E-03	1.47E-03	0.49
P01859	IGHG2	Ig gamma-2 chain C region	7.81E-03	7.03E-04	0.63
P08603	CFH	Complement factor H	8.18E-03	1.93E-03	0.67
P01860	IGHG3	Ig gamma-3 chain C region	8.24E-03	9.29E-04	0.40
P02749	APOH	Beta-2-glycoprotein 1	9.01E-03	1.06E-03	0.14
P00740	F9	Coagulation factor IX	1.21E-02	2.07E-03	1.51
P80108	GPLD1	Phosphatidylinositol-glycan-specific phospholipase D	1.45E-02	2.51E-03	1.63
P00734	F2	Prothrombin	1.61E-02	1.21E-02	0.63
P01717	IGLV3-25	Immunoglobulin lambda variable 3-25	1.69E-02	2.72E-02	2.11
O95445	APOM	Apolipoprotein M	1.69E-02	1.88E-02	0.55
Q06033	ITIH3	Inter-alpha-trypsin inhibitor heavy chain H3	2.37E-02	9.87E-03	1.50

A0A0C4DH31	IGHV1-18	Immunoglobulin heavy variable 1-18	2.48E-02	1.44E-02	0.58
P02774	GC	Vitamin D-binding protein	2.51E-02	2.25E-02	0.42
O43866	CD5L	CD5 antigen-like	2.57E-02	4.20E-03	0.33
P25311	AZGP1	Zinc-alpha-2-glycoprotein	3.97E-02	7.85E-03	0.31
P01780	IGHV3-7	Immunoglobulin heavy variable 3-7	4.92E-02	2.98E-02	1.96

*Ratios of means calculated as first experimental group divided by second experimental group according to the sequence displayed in the table titles.

Supplementary Table S5: Comparison of different machine learning algorithms.

***p*-value threshold 0.05** *(A)* 24 proteins, *(B)* 12 proteins*

	(A) **AC vs. COPD**		***(B)*** **AC with COPD vs. COPD**	
	**AUC[§]**	**PRAUC[§]**	**AUC**	**PRAUC**
**Logistic regression**	0.830	0.684	0.880	0.801
**Linear discriminant analysis**	0.843	0.818	0.748	0.657
**SVM with linear kernel**	0.858	0.851	**0.889**	**0.836**
**SVM with polynomial kernel**	0.876	0.869	0.880	0.805
**Random forest**	**0.902**	**0.880**	0.875	0.823

***p*-value threshold 0.1** *(A)* 36 proteins, *(B)* 20 proteins

	(A) **AC vs. COPD**		***(B)*** **AC with COPD vs. COPD**	
	**AUC**	**PRAUC**	**AUC**	**PRAUC**
**Logistic regression**	0.748	0.168	0.769	0.146
**Linear discriminant analysis**	0.840	0.816	0.743	0.650
**SVM with linear kernel**	0.856	0.847	**0.910**	**0.876**
**SVM with polynomial kernel**	0.869	0.851	0.900	0.817
**Random forest**	**0.912**	**0.894**	0.889	0.838

***p*-value threshold 0.2** *(A)* 66 proteins, *(B)* 41 proteins

	(A) **AC vs. COPD**		***(B)*** **AC with COPD vs. COPD**	
	**AUC**	**PRAUC**	**AUC**	**PRAUC**
**Logistic regression**	0.696	0.193	0.750	0.159
**Linear discriminant analysis**	0.837	0.809	0.736	0.641
**SVM with linear kernel**	0.856	0.847	**0.908**	0.865
**SVM with polynomial kernel**	0.869	0.851	0.890	0.805
**Random forest**	**0.935**	**0.928**	0.906	0.**875**

***p*-value threshold 0.3** *(A)* 87 proteins, *(B)* 45 proteins

	(A) AC vs. COPD		*(B)* AC with COPD vs. COPD	
	**AUC**	**PRAUC**	**AUC**	**PRAUC**
**Logistic regression**	0.713	0.189	0.705	0.170
**Linear discriminant analysis**	0.837	0.809	0.732	0.639
**SVM with linear kernel**	0.856	0.847	**0.908**	0.865
**SVM with polynomial kernel**	0.869	0.851	0.890	0.805
**Random forest**	**0.916**	**0.913**	0.903	**0.871**

***p*-value threshold 0.5** *(A)* 149 proteins, *(B)* 68 proteins

	(A) AC vs. COPD		*(B)* AC with COPD vs. COPD	
	**AUC**	**PRAUC**	**AUC**	**PRAUC**
**Logistic regression**	0.692	0.195	0.657	0.183
**Linear discriminant analysis**	0.835	0.806	0.720	0.627
**SVM with linear kernel**	0.856	0.851	0.908	**0.865**
**SVM with polynomial kernel**	0.869	0.811	0.890	0.805
**Random forest**	**0.917**	**0.905**	**0.914**	0.852

***p*-value threshold not applied** *(A)* 194 proteins, *(B)* 194 proteins

	(A) AC vs. COPD		*(B)* AC with COPD vs. COPD	
	**AUC**	**PRAUC**	**AUC**	**PRAUC**
**Logistic regression**	0.676	0.199	0.611	0.191
**Linear discriminant analysis**	0.835	0.806	0.709	0.620
**SVM with linear kernel**	0.856	0.847	0.908	0.865
**SVM with polynomial kernel**	0.869	0.851	0.890	0.805
**Random forest**	**0.921**	**0.912**	**0.916**	**0.882**

* For feature selection, the *p*-value threshold was varied; a correlation-cut off of 0.7 was constantly applied. The resulting numbers of considered proteins are displayed next to the table captions.
§ Cross-validated area under the curve (AUC) and area under the precision recall curve (PRAUC) were used for model optimization.

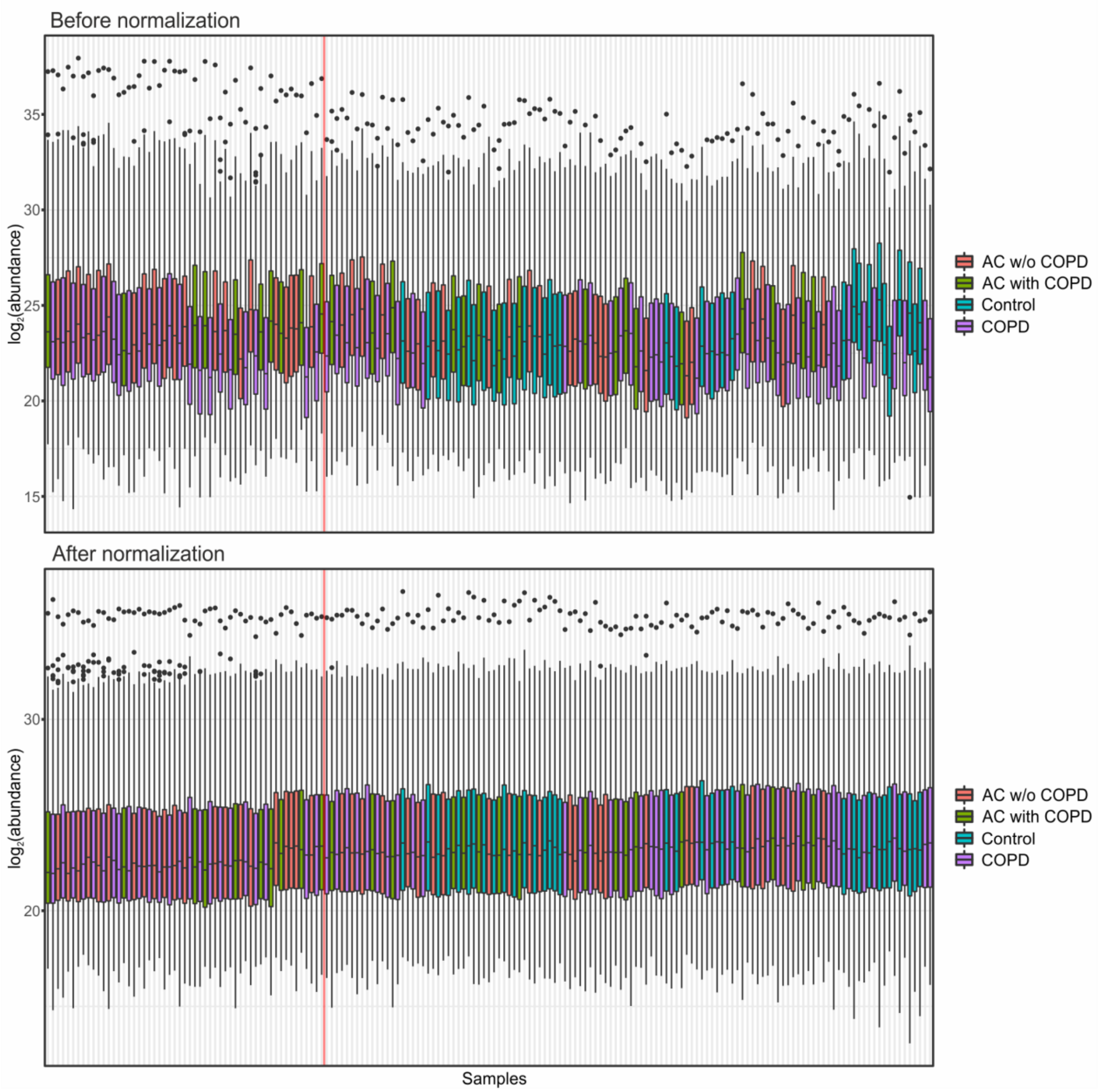

Supplementary Figure S1: Boxplot representation of protein intensities before and after normalization. Red line indicating the transition between first batch (left) and second batch (right). Boxes indicate the 25% - 75% interquartile range (IQR) with the median displayed as horizontal line. Whiskers extend to 1.5 x IQR. Outliers displayed as individual data points.

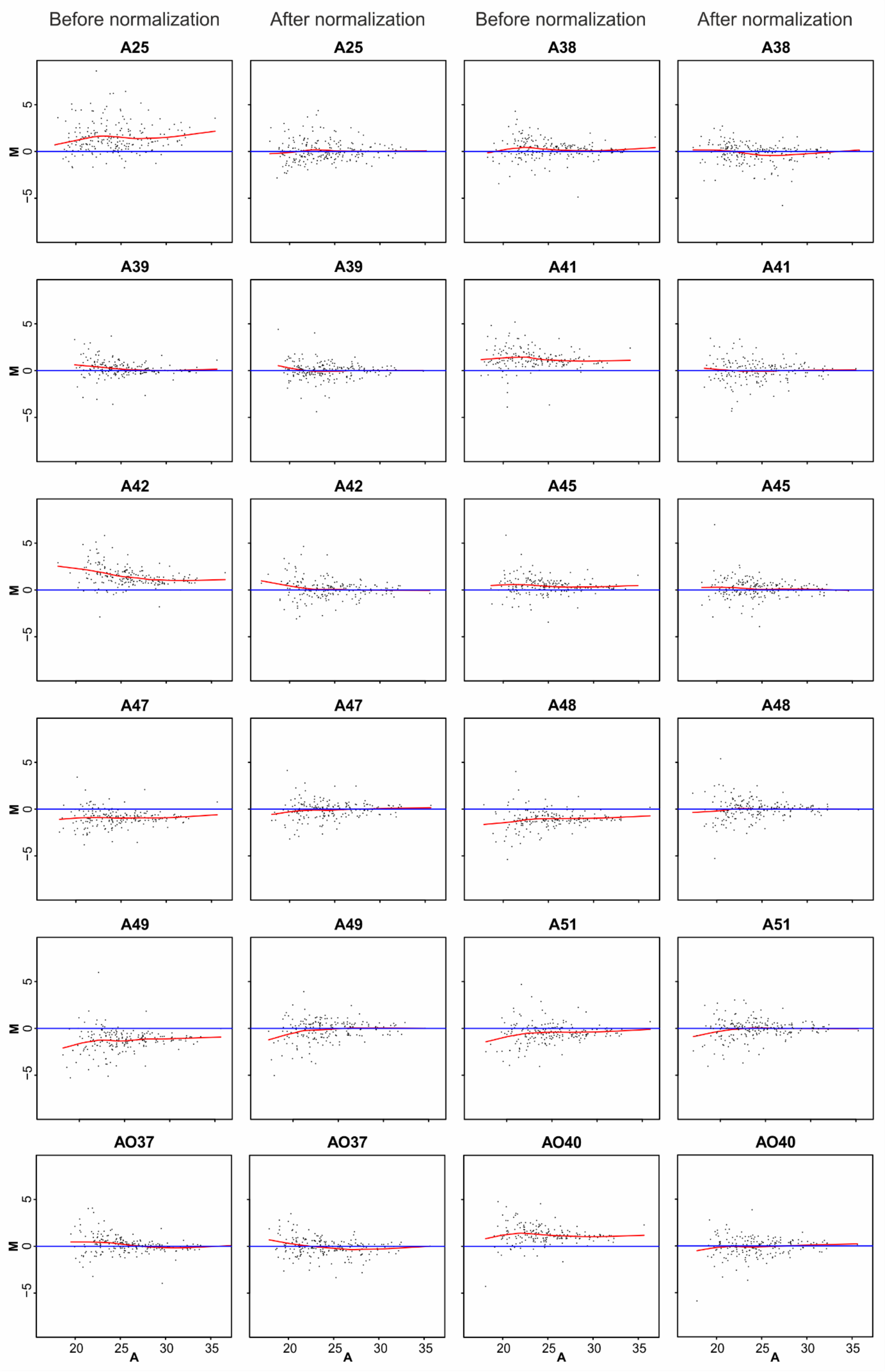

Before normalization
After normalization
Before normalization
After normalization
A25
A25
A38
A38
A39
A39
A41
A41
A42
A42
A45
A45
A47
A47
A48
A48
A49
A49
A51
A51
AO37
AO37
AO40
AO40
M
A
-5
0
5
20
25
30
35

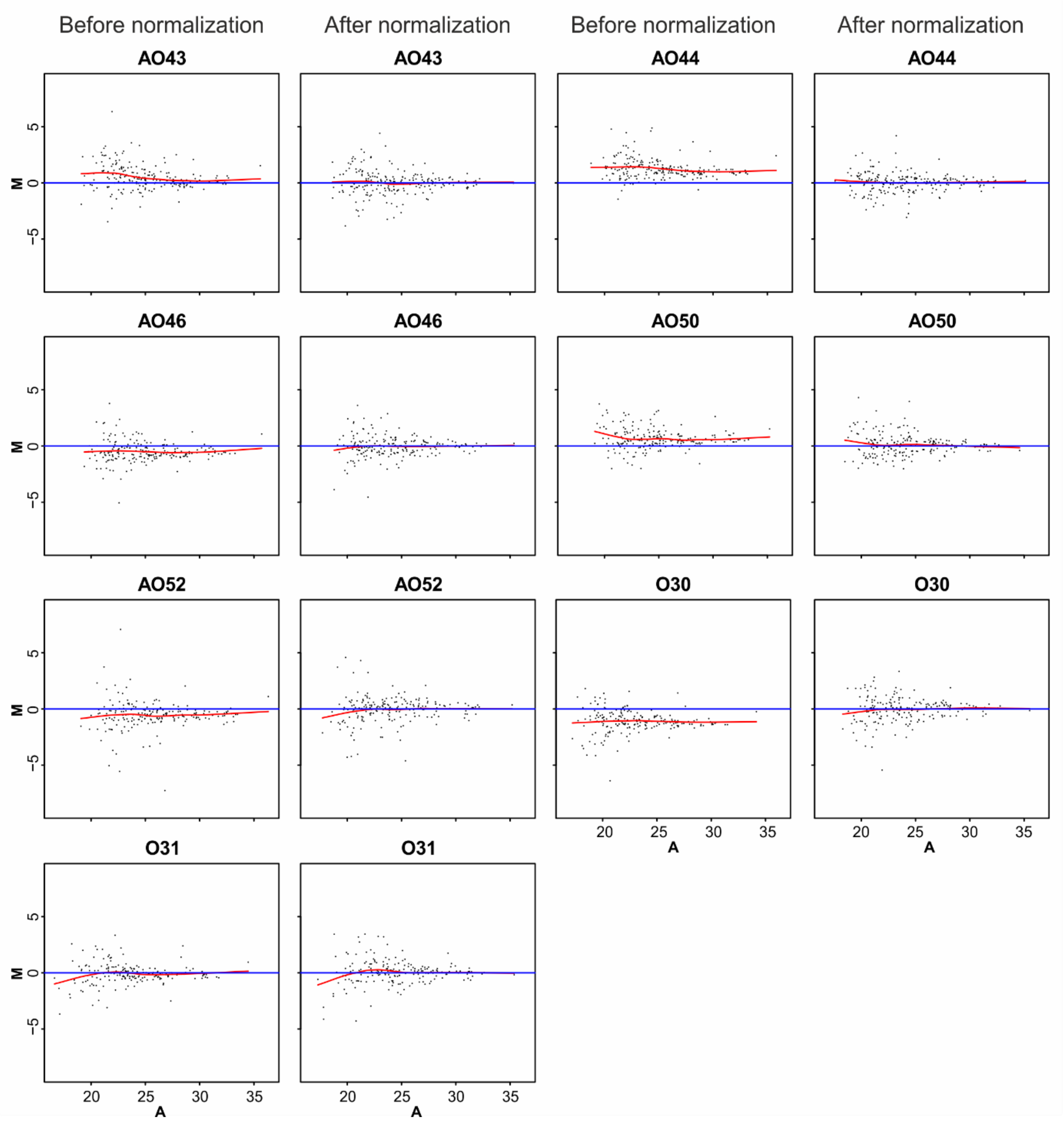

Supplementary Figure S2: MA Plots representing the two measurements of samples that were doubly measured in both batches. The MA Plots are shown before and after the batch normalization for each patient. On the x-axis, the mean (A = average) and on the y-axis the difference (M = minus) between the log2-transformed intensities are shown. Each point represents one protein with low abundant proteins on the left and high abundant proteins on the right of the plots. At the top or bottom, there are proteins which strongly change between the compared measurements. Proteins that do not show prominent changes after normalization stay around y = 0 (blue line). The red line is the local regression line fitted to the point cloud. Ideally, this line would be equal to the blue horizontal line at y = 0. If the red line is shifted from the blue line, this indicates a technical bias. Before normalization most samples show a high deviation of the local regression line from the blue line at y = 0, which is explainable by the technical variation between the two batches. After normalization this bias is greatly reduced and the local regression line is very close to y = 0. By this we can conclude that the batch normalization worked as intended and removed the differences caused by the measurements in two batches.

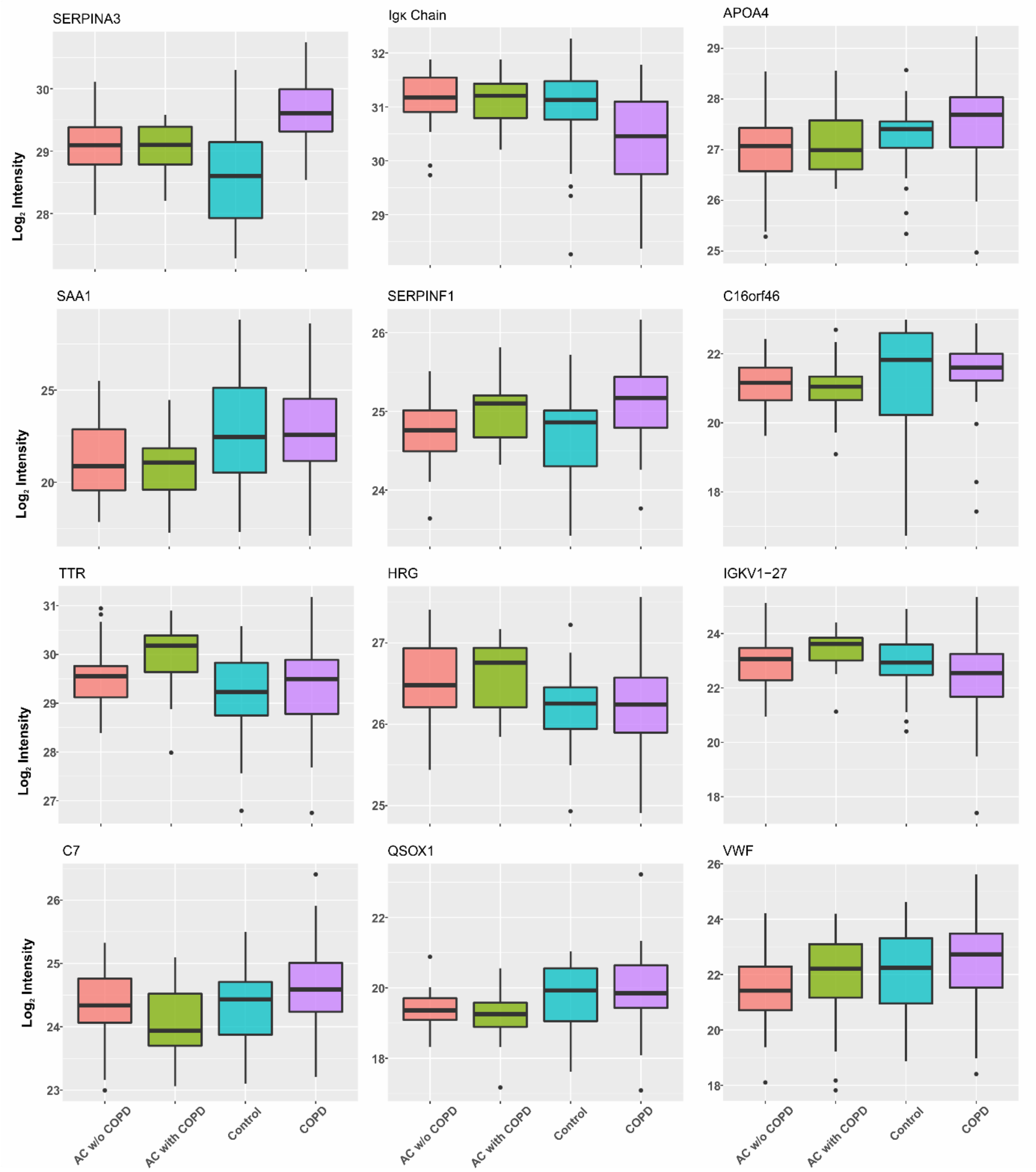

Supplementary Figure S3: Boxplot representation of normalized protein intensities shown for selected biomarker candidates that were highlighted in the manuscript. Boxes displayed separately for the analyzed patient groups, which are also indicated by colors. Boxes indicate the 25% - 75% interquartile range (IQR) with the median displayed as horizontal line. Whiskers extend to 1.5 x IQR. Outliers displayed as individual data points.
